# Real-World Evaluation of the Safety and Efficacy of Surgi-ORC® in Orthopedic Surgeries: A Multicenter Prospective Study

**DOI:** 10.7759/cureus.78616

**Published:** 2025-02-06

**Authors:** Piyush Patel, Diksha Sharma, Bhavin Trivedi, Shahid Karatela

**Affiliations:** 1 Quality, Aegis Lifesciences Pvt. Ltd., Ahmedabad, IND; 2 Research and Development, Aegis Lifesciences Pvt. Ltd., Ahmedabad, IND; 3 Regulatory Affairs, Aegis Lifesciences Pvt. Ltd., Ahmedabad, IND; 4 Regulatory Affairs and Quality Assurance, Aegis Lifesciences Pvt. Ltd., Ahmedabad, IND

**Keywords:** haemostasis, orthopedic surgery, oxidized regenerated cellulose (orc), surgi-orc® fibril, surgi-orc® non-woven/snow, surgi-orc® original, surgi-orc® powder, surgi-orc® standard

## Abstract

Aim

Achieving adequate hemostasis is a critical aspect of surgical practice, especially in orthopedics, where bleeding can impact visibility and patient outcomes. Surgi-ORC® (Aegis Lifesciences Pvt. Ltd., Ahmedabad, India), an oxidized regenerated cellulose hemostatic agent, was evaluated for its safety, efficacy, biocompatibility, and clinical performance in real-world patients undergoing orthopedic surgeries.

Method

This prospective observational study included patients undergoing upper limb, lower limb, or axial skeletal orthopedic surgeries. Blood loss, time to hemostasis (TTH), and surgeon-rated performance parameters such as ease of application, conformation to tissue, and preparation were recorded to assess the performance of Surgi-ORC® at predefined time points. Biocompatibility assessments were conducted through in vitro and in vivo tests, including reactivity, sensitization, systemic toxicity, tissue response, and hemolytic potential.

Results

The study involved 59 patients undergoing orthopedic surgeries using Surgi-ORC® variants: Powder (n=4), Knit (n=5), Original/Standard (n=4), Fibril (n=42), and Non-Woven/Snow (n=4). Hemostasis was achieved in 100% of cases within ≤ five minutes, with a mean TTH of 1.95 minutes (Surgi-ORC® Original/Standard). Powder demonstrated the lowest mean TTH across all surgeries, averaging 0.83 to 1.08 minutes. Surgeon feedback highlighted excellent handling, ease of application, and conformance to tissue without sticking/adherence to surgical gloves/surgical instruments. No infections, adverse events, or complications occurred over 60 days, and all patients showed stable recoveries. The surgeon confirmed complete absorption by day two.

Conclusion

Surgi-ORC® is a reliable and effective hemostatic agent for orthopedic surgeries. Its ease of use, handling characteristics, ease of placing, stability upon placement, and safety profile make it a preferred choice among surgeons.

## Introduction

Bone fractures present significant challenges for orthopedic surgeons and often result from trauma, osteoporosis, or bone cancer [1]. According to the Global Burden of Disease Study (GBD), the global number of new fracture cases was estimated to be 178 million in 2019, indicating an increase of 33.4% since 1990. The top anatomical sites reported were fractures of the patella, tibia, fibula, or ankle; radius or ulna, or both; and clavicle, scapula, or humerus [2]. Complex fractures, particularly those involving multiple fragments, require intricate surgical interventions and are associated with poor clinical outcomes. Some of the most challenging fractures affect the facial bones, foot, ankle, clavicle, and tibia. In emergency departments, distal radius fractures, often accompanied by fractures of the distal ulna, are the most common upper extremity injuries. Distal radius fractures are especially prevalent, with approximately 20% of cases requiring surgical intervention [3]. Notably, the need for surgery is rising among younger individuals, with 45% of fractures in patients under 25 years old and 37.5% in those aged between 25 and 30 years requiring surgery [4,5]. Other significant fractures include clavicle fractures, accounting for 2.6% to 5% of adult fractures, and proximal tibial fractures, representing about 1% of all fractures [6]. Proximal tibial fractures predominantly affect individuals aged between 40 and 60 years [7]. According to a study by Khan et al., it was concluded that there is an association between type of fracture and age [8]. Distal tibia fractures, often caused by traffic accidents or falls, represent 3% to 10% of tibial fractures and 1% of lower extremity fractures [9].

Surgical management of these fractures typically involves open reduction and internal fixation (ORIF), where the fracture site is exposed, reduced, and stabilized with devices like wires, plates, and screws [10]. However, perioperative blood loss is a significant challenge in orthopedic surgery. Excessive bleeding during and after surgery is associated with complications such as wound issues, infections, delayed rehabilitation, prolonged hospital stays, and increased healthcare costs [11]. Blood loss is a risk during orthopedic surgery, with an estimated blood loss of 1,188-1,651 mL in total knee replacement [12]. The challenge faced in interpreting bleeding after orthopedic surgery is using different criteria to define bleeding [13]. To mitigate these issues, hemostatic agents are widely used in orthopedic surgeries to control perioperative bleeding. These agents can be classified into bone wax, hemostasis-osteogenesis materials, and biodegradable materials. Oxidized regenerated cellulose (ORC) is a biodegradable, natural plant-based topical hemostatic agent. Surgi-ORC® (Aegis Lifesciences Pvt. Ltd., Ahmedabad, India) achieves hemostasis through a combination of physical and biochemical mechanisms. Upon contact with blood, Surgi-ORC® absorbs fluid and transforms into a brownish or black gelatinous mass. This mass acts as a physical matrix that facilitates platelet adhesion and aggregation, leading to the formation of a platelet-fibrin plug. Simultaneously, the swelling of Surgi-ORC® generates pressure on the surrounding tissue, further contributing to hemostasis. Additionally, the acidic environment created by ORC helps stabilize the clot and inhibits fibrinolysis, ensuring effective bleeding control. It has emerged as a novel therapeutic approach for controlling surgical bleeding due to its safety, ease of use, and proven efficacy [14].

Oozing and spot bleeding during surgery are diffuse and hard to locate, making management difficult. Small vessel bleeding, like capillaries, is more challenging to control than bigger vessel bleeds. The widespread bleeding makes it difficult to use typical hemostatic procedures like ligation or cauterization, and anticoagulant treatment or underlying disorders can worsen coagulation difficulties. Spot bleeding can potentially lengthen surgery and raise the risk of infections and hematomas. Surgi-ORC® presents an effective approach for controlling oozing and spot bleeding during surgical interventions, tackling a prevalent issue in surgical practices.

Surgi-ORC® is an efficient and adaptable product utilized in surgical procedures to control bleeding, with distinctive characteristics such as plasticity, consistency, and manageability. Multiple variations of ORC, such as Surgi-ORC® Original/Standard, Surgi-ORC® Knit, Surgi-ORC® Fibril, Surgi-ORC® Non-woven/Snow, and Surgi-ORC® Powder, have been designed to enhance hemostasis and facilitate prompt bleeding control in various surgical environments. Surgi-ORC® Original/Standard and Knit offer superior strength for heavier bleeding. The flexibility of ORC allows it to conform effortlessly to complex tissue surfaces, ensuring a secure fit and superior coverage of the surgical site. Its consistency ensures the maintenance of structural integrity and efficacy throughout the process, aiding hemostasis and enhancing clot formation. Surgi-ORC® reliably maintains its mechanism of action, proficiently controlling hemorrhage, including localized bleeding. The material's manipulability enhances its value, allowing surgeons to maneuver, incise, and position it precisely at the intended site, even in complex or confined surgical settings.

The flexible and lightweight design of Surgi-ORC® Fibril and Non-Woven/Snow enhances its usability by allowing for effortless removal with forceps, facilitating precise handling, cutting, and placement by surgeons in targeted areas, even within difficult or restricted surgical environments. Surgi-ORC® Fibril, Non-Woven/Snow, and Powder facilitate the adaptation to uneven wound topographies and anatomically intricate regions, guaranteeing an exact fit for complicated surgical sites. Surgi-ORC® Fibril consists of seven layers of fibrillose material that guarantee thorough integration and absorption throughout the body. This approach is optimal for intricate orthopedic surgeries when quick and efficient hemostasis is essential.

Surgi-ORC®’s low pH of one to three is attributed to its antibacterial activity, which results in a lower chance of postoperative infection. In-house pH and in vitro antibacterial study data of Surgi-ORC® are established and available. Surgi-ORC® powder is prefilled in an applicator/bellow bottle to be dispensed on the target bleeding site in dry form. Surgi-ORC® is used in many thoracic, neurosurgical, general, gynecological, and urological surgeries. However, there is limited literature on the use of ORC in orthopedic surgery, particularly in cases involving upper and lower limb axial skeleton surgeries. This study aims to evaluate the hemostatic efficacy and safety of Surgi-ORC® in orthopedic surgery, addressing a gap in current research [15] and expanding its documented applications in orthopedics.

## Materials and methods

About Surgi-ORC^®^

Five variants of Surgi-ORC®, namely Original/Standard, Knit, Fibril, Non-woven/Snow, and Powder, were used in this study (Appendix A). Each unit of Surgi-ORC® was individually packaged in a double-wrapping single sterile packaging system, with adequate data on labels for ease of use. Comprehensive training materials, including an instruction for use (IFU) guide and product brochures, were supplied to the intended users to ensure proper handling and application, and training was also provided to physicians or surgeons. These resources guarantee that the instructions are explicit and attainable, facilitating safe and efficient implementation in surgical procedures. Surgi-ORC® was encased in a double-wrapping single sterile barrier system. Additionally, the product is designed to remain non-adherent to its packaging, ensuring easy retrieval and application without the risk of material loss or contamination. This packaging guarantees the product's sterility during transport and storage, preserving the necessary sterility until utilized. Surgi-ORC® is engineered to preserve its integrity at the bleeding site during application and exhibits no tearing, crumbling, or shrinking. Furthermore, the product exhibits a uniform texture, enhancing its ease of application and excellent adherence to the surgical site. Surgi-ORC® has significant tensile strength in wet and dry conditions, guaranteeing durability and stability, even in complex surgical events, ensuring reliable performance and optimal hemostasis during the surgical procedure.

Study design

This was a prospective, open-label, multicenter, single-arm observational study conducted after review and approval of the ethics committees of Jasleen Hospital, Nagpur India (approval number: AL/PMCFP/ORC/RO) and Shri Shankaracharya Institute of Medical Sciences, Durg, India (approval number: IEC/SSIMS /RP/2024/04). It is registered with the Clinical Trials Registry of India (Registration number: CTRI/2024/02/062445-ORTH/001). The study was conducted for 60 days in accordance with the International Organization for Standardization (ISO) 14155:2020 standards and the International Conference on Harmonization's Good Clinical Practice (ICH-GCP) guidelines.

Study population

The study included participants aged 18 years and above who underwent elective or emergency orthopedic surgeries where intraoperative bleeding is expected and the hemostatic agent is required. The inclusion criteria required patients to be willing to participate in the study, who underwent orthopedic surgery with anticipated intraoperative bleeding necessitating a hemostatic agent, and who demonstrated readiness to comply with all study-related requirements. The exclusion criteria included patients with known hypersensitivity to cellulose; patients who are known or suspected to be pregnant or lactating; those with an active infection at the surgical site; patients diagnosed with a bleeding disorder such as thrombocytopenia, thrombasthenia, hemophilia, or von Willebrand disease; and patients simultaneously enrolled in another clinical trial.

Data acquisition

Clinical data were collected from preoperative, operative, and three postoperative follow-ups, and it was recorded in paper case report forms.

Outcomes

The performance of Surgi-ORC® was established based on the primary and secondary endpoints, including the determination of time to hemostasis (TTH) on day 0 by the proportion of wounds achieving hemostasis at every two minutes, five minutes, and 10 minutes after the application; hemostatic handling characteristics through the surgeon's questionnaire on day zero; liquefaction of the device post procedure through radiological examination after two days of its implantation; and complete absorption of the device through radiological examination after 28 days of its implantation. The safety of Surgi-ORC® was assessed by evaluating the rate of adverse reactions due to the implantation of Surgi-ORC® (such as postoperative infections, foreign body reactions, adhesion formation, or incomplete resorption of Surgi-ORC®) from day 0 to 60 days post procedure.

Follow up

There were three follow-ups after the surgery: day two, day 28, and day 60. On day two, a radiological examination was carried out to determine the start of absorption. On day 28, a radiological examination was carried out to determine the complete absorption of Surgi-ORC®. A radiological examination for complete absorption of Surgi-ORC® was only done on day 28 for subjects where confirmation of absorption was not available earlier during the radiological examination on day two. Laboratory tests were performed as per the recommendation of the site investigator. Adverse events were recorded for all three follow-up periods.

Ethical statement

The study protocol was reviewed and approved by the participating site's ethics committee, ensuring compliance with ethical standards. The study was conducted as per the Indian Council of Medical Research (ICMR) Guidelines for Biomedical Research on Human Subjects, Schedule Y (Amended Version 2013), and ICH-GCP guidelines E6 (R1) and by the ethical principles that have their origin in the Declaration of Helsinki (Fortaleza, Brazil, October 2013) and other applicable regulatory requirements, as well as in compliance with the approved protocol.

Statistical analysis

Demographic characteristics were analyzed using descriptive statistics. Categorical variables were expressed as frequencies and percentages, while continuous variables were summarized by count, mean, and standard deviation. Regardless of whether they were explicitly mentioned in the statistical plan, all relevant clinical assessments were listed and, where applicable, summarized using descriptive statistics based on categorical data of interest. The Chi-square test was utilized to compare categorical data. Independent samples of the Kruskal-Wallis test and Bonferroni-corrected pairwise comparisons were used to compare products' ability to produce hemostasis. Results were further visualized using graphs and statistical tests. All statistical analyses were done using IBM SPSS Statistics software, version 27.1 (IBM Corp., Armonk, NY). A p-value < 0.05 was considered statistically significant.

## Results

Demographics and preoperative outcomes

The study included 59 subjects with a mean (SD) age of 43.22 (18.12) years (Table 1). The most common cause of orthopedic surgery was lower limb fracture, accounting for 64.40%, which comprised hip, knee, ankle, foot, tibia, fibula, and femur fractures. This was followed by upper limb fractures (32.20%), which included shoulder, wrist, hand, humerus, forearm, clavicle fracture, and axial skeleton fractures (3.38%), encompassing cervical spine and pelvic fractures.

**Table 1:** Demographic and bleeding characteristics across the population

	Overall group (N=59)
Patient characteristic	
Age at consent, years	
Mean (SD)	42.22 (18.28)
Median (range)	41 (19, 89)
Gender, n(%)	
Male	41 (69.5%)
Female	18 (30.5%)
Target bleeding characteristic, n(%)	
Mild	21 (35.6%)
Moderate	38 (64.4%)
Size (area), cm^2^	
Mean (SD)	26.26 (17.51)
Median (range)	20 (3, 80)

Study outcomes

A total of 59 patients were treated with different variants of the subject device used, and patients were categorized based on the bleeding grade. While most of the patients did not require conventional methods to stop the bleeding, electrocoagulation and mechanical methods were used in a few patients. Total blood loss during the procedure was measured in all participants. The mean blood loss was 60.96 mL (SD = 32.55), ranging from 15 mL to 200 mL. The median blood loss was 55 mL (interquartile range (IQR): 25), and no patient experienced blood loss exceeding 200 mL. Surgi-ORC® Powder (n=4), Knit (n=5), Original/Standard (n=4), Fibril (n=42), and Non-Woven/Snow (n=4) were used for hemostasis. The study's primary endpoint was the determination of TTH and the proportion of wounds achieving hemostasis every two minutes, five minutes, and 10 minutes after the application of Surgi-ORC®. Hundred percent of the patients achieved hemostasis within ≤ five minutes. The average TTH using the Surgi-ORC® reported in the patients was 1.95 minutes (Table 2).

**Table 2:** Comparison of mean time to hemostasis (TTH) across different product types and surgery type

Product type	Mean TTH (minutes)			Average
	Upper limb (SD=0.46)	Lower limb (SD=0.74)	Axial skeleton (SD=0.51)	
Surgi-ORC^®^ Original/Standard	2.25	2.44	1.16	1.95
Surgi-ORC^®^ Knit	2.17	2.50	2.18	2.28
Surgi-ORC^®^ Fibril	1.67	2.18	1.16	1.67
Surgi-ORC^®^ Non-Woven/Snow	1.76	1.67	1.16	1.53
Surgi-ORC^®^ Powder	1.08	0.71	0.83	0.87

Product type and TTH

**Figure 1:**
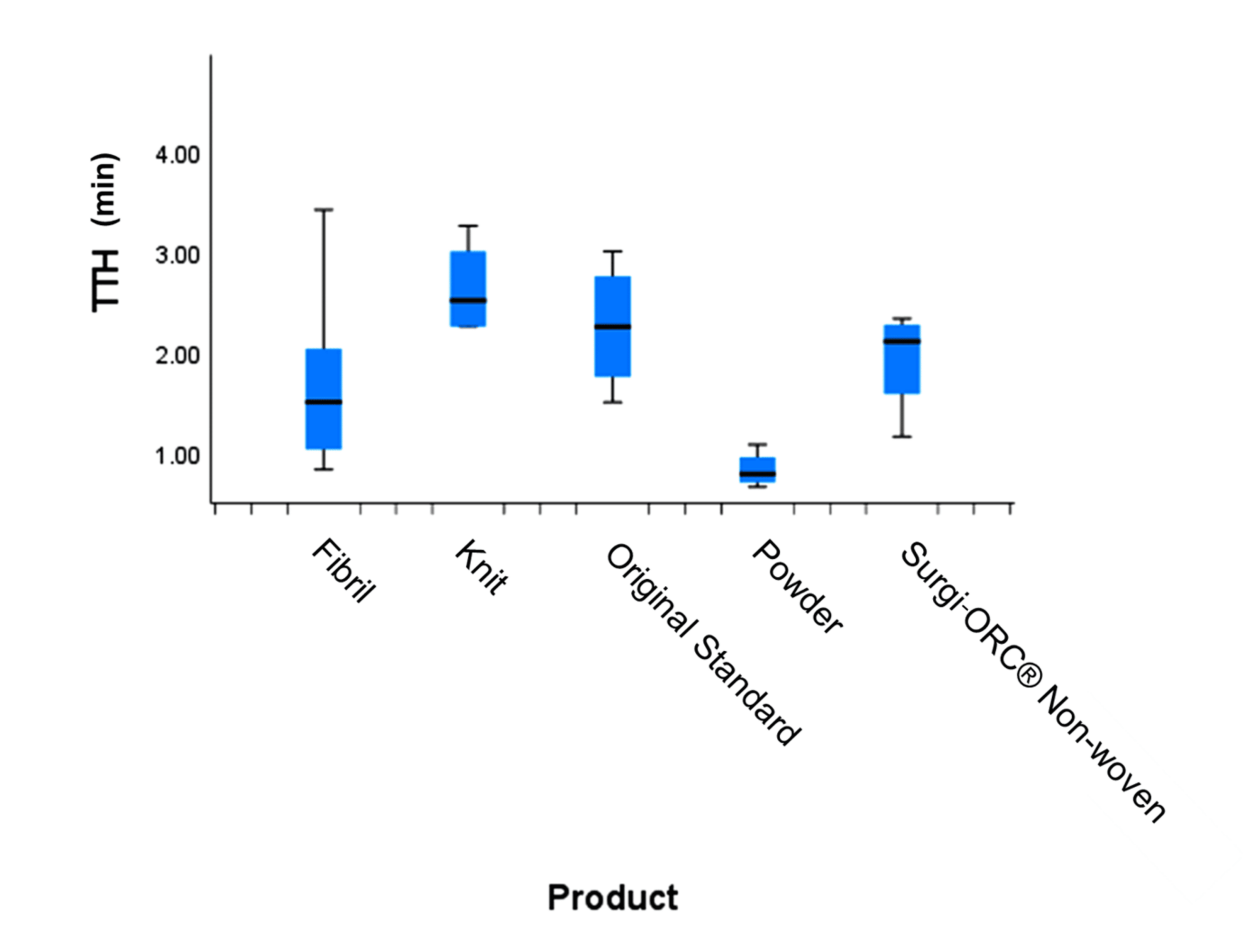
Comparison of time to hemostasis (TTH) across products min: minutes

Time to hemostasis was compared across five distinct products using the independent samples Kruskal-Wallis test (Figure 1). The Kruskal-Wallis test, followed by Bonferroni-corrected pairwise comparisons, revealed a significant difference between Knit and Powder (p-value = 0.015). Knit (2.2 min) demonstrated a longer time to achieve hemostasis than Powder (0.87 min). No other statistically significant differences were observed between the products.

Surgery and degree of bleeding

**Figure 2:**
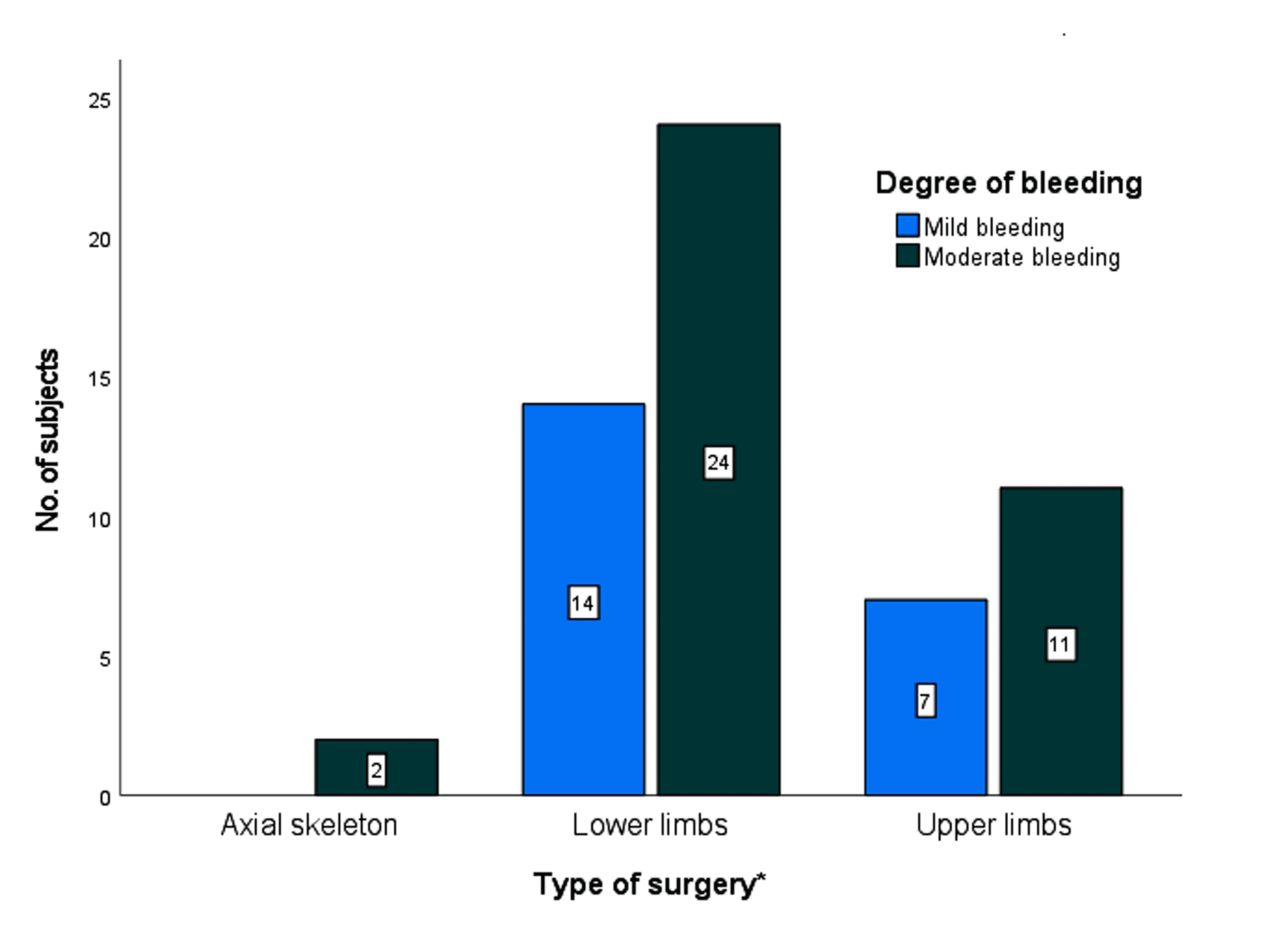
Bleeding characteristics according to the type of surgery *Upper limbs include shoulder, wrist, hand, humerus, forearm, and clavicle fractures; Lower limbs include hip, knee, ankle, tibia, fibula, foot, and femur fractures. The axial skeleton includes the cervical spine and pelvic fractures

Figure 2 shows the distribution of subjects according to the type of surgery performed and the related degree of bleeding. The chi-square value for the degree of bleeding and type of surgery was found to be 1.946, with a p-value of 0.549, indicating no statistically significant association between the two variables.

Surgery and TTH

**Figure 3:**
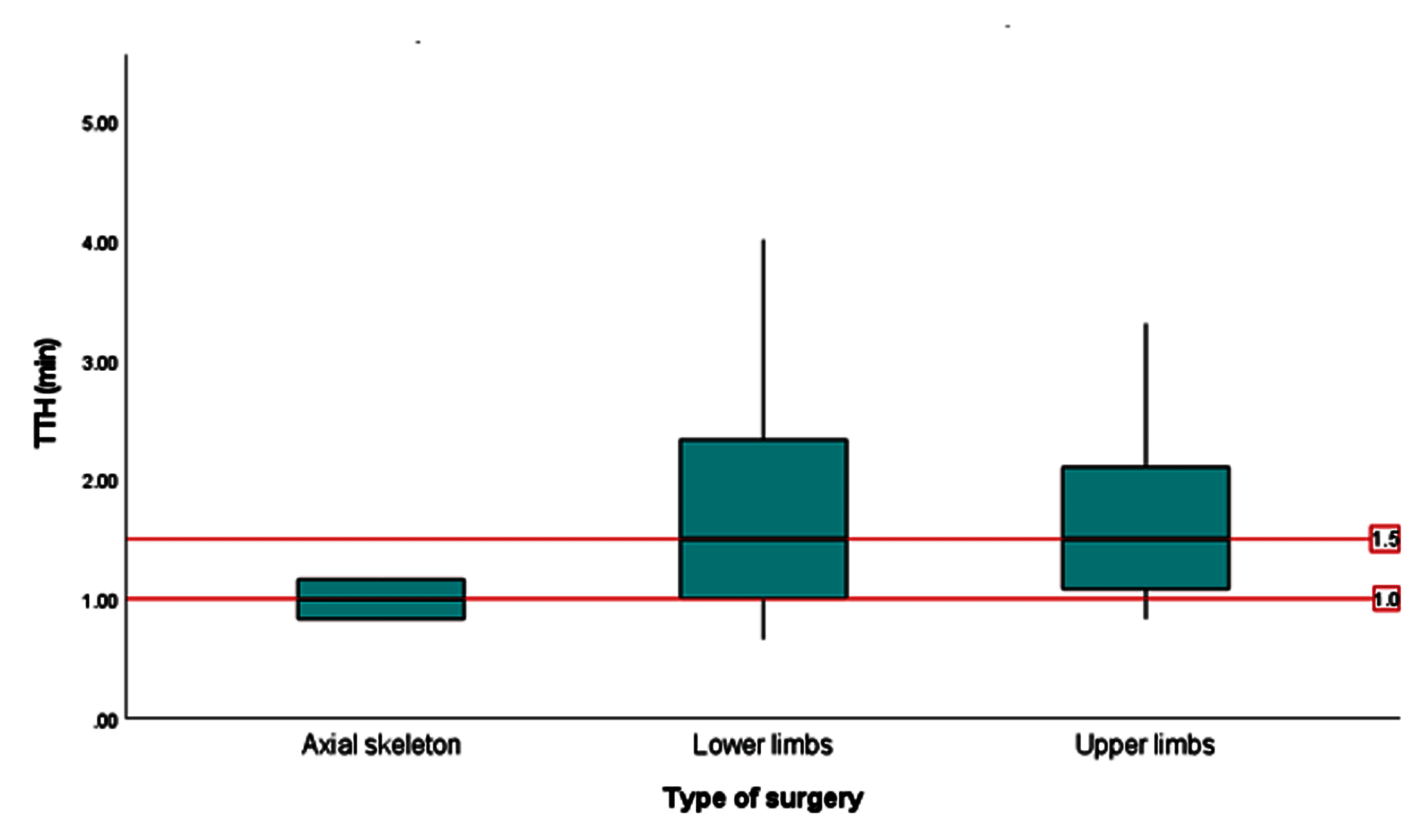
Boxplot of time to hemostasis (TTH) by type of surgery min: minutes

Analysis of variance (ANOVA) was performed to compare TTH with the type of surgery; the result indicated no statistically significant association between these two variables, with an F value of 0.794 (p-value = 0.457). A boxplot analysis (Figure 3) compared the TTH across three surgeries: axial skeleton, lower limbs, and upper limbs. The median TTH for axial skeleton surgeries was the lowest at approximately 1.0 minutes. The IQR was narrow, ranging from about 0.8 to 1.2 minutes, indicating low variability. In contrast, lower limb surgeries had the highest median TTH at approximately 2.5 minutes. The IQR was wider, ranging from about 1.8 to 3.5 minutes, indicating higher variability and more spread in the data. Upper limb surgeries had a median TTH of around two minutes, falling between the axial skeleton and lower limb surgeries. The IQR for upper limb surgeries ranged from about 1.5 to 2.5 minutes, showing moderate variability.

Surgery, product type, and TTH

**Figure 4:**
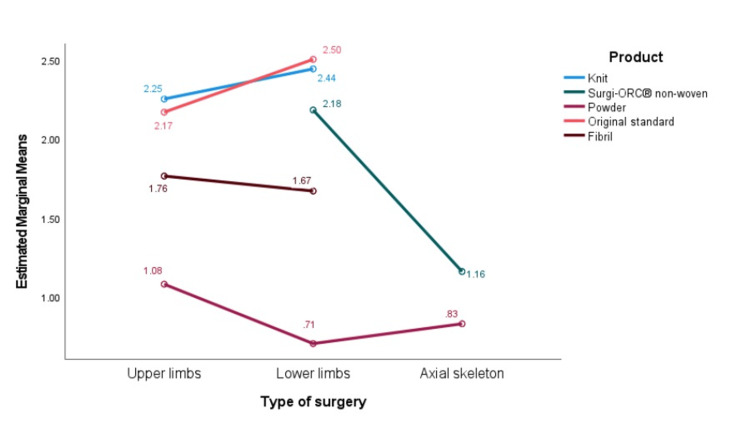
Comparison of time to hemostasis (TTH) across different products in various surgery

Two-way ANOVA was performed (Figure 4) to assess which product performed better in a specific surgery context, which aids in deciding product usage based on the type of surgery. The calculated marginal means of TTH were compared between various products utilized in the axial skeleton, lower limb, and upper limb procedures. The data revealed significant differences in TTH across various products and surgical techniques. Surgi-ORC® Original/Standard Product had the highest mean TTH for upper limb surgery at 2.25 minutes, followed by Knit (2.17 minutes), Non-woven/Snow (1.76 minutes), Fibril (1.67 minutes), and Powder (1.08 minutes). Surgi-ORC® Knit had the highest mean TTH in lower limb surgery at 2.50 minutes, followed by the Original/Standard Product at 2.44 minutes, Fibril at 2.18 minutes, Non-woven/Snow at 1.67 minutes, and Powder at 0.71 minutes at the lowest mean TTH. For axial skeleton surgery, Surgi-ORC® Knit had the highest mean TTH at 2.18 minutes, while the Original/Standard product, Non-woven/Snow, and Fibril all showed similar mean TTHs of 1.16 minutes, and Powder had a mean TTH of 0.83 minutes. Overall, Surgi-ORC® Powder consistently demonstrated the lowest mean TTH across all surgeries, suggesting it might be the most efficient variant for reducing TTH.

In contrast, Surgi-ORC® Knit tended to have the highest TTH, especially in lower limbs and axial skeleton surgeries. No postoperative drainage was required for any subjects. On day two, follow-up liquification of Surgi-ORC® was confirmed by X-ray examination. There was no need for dressing reinforcement in the subject population. No infection was observed on day 28 of follow-up. No adverse events, including infection, adhesion formation, or foreign body reaction, were observed during the study. At the 60-day follow-up post surgery, the wound exhibited satisfactory healing.

Radiological examination of Surgi ORC^®^ post implantation 

**Figure 5:**
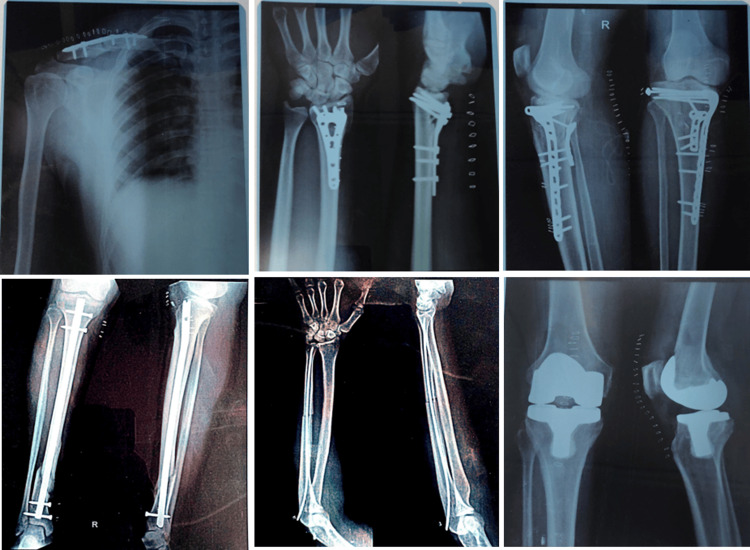
Post-surgical X-ray examination shows no traces or residue of Surgi-ORC® on day two

One of the safety and performance endpoints of the study was to determine the complete absorption of Surgi-ORC® through radiological examination after the second and 28 days of its implantation. The product was completely absorbed in all 59 patients on postoperative day two and evidenced through radiological examination as shown in Figure 5.

Usability evaluation of Surgi-ORC^®^ by surgeons

The evaluation of Surgi-ORC® performance based on 59 surgeons' feedback demonstrated high satisfaction across multiple characteristics (Table 3). Thirty-two surgeons rated ease of application as "excellent," and 27 rated it as "good". The product's conformation to tissue surfaces was rated as "excellent" by 59% of surgeons, while 23 (38.9%) rated it as "good," and only one (1.6%) rated it as "poor". Regarding ease of delivery to the wound site, 37 (62.7%) of surgeons rated it as "excellent" and 22(37.3%) as "good," with no "poor" ratings observed. Similarly, the ease of preparation for use was rated "excellent" by 36 (61%) of surgeons and "good" by 39%. These findings highlight the product's practical effectiveness and its positive user experience in surgical environments.

**Table 3:** Evaluation of the hemostatic product's (Surgi-ORC®) performance by surgeons

Characteristic	Excellent	Good	Poor
Ease of application of the product at the bleeding site	32 (54.2%)	27 (45.8%)	-
Conformation of the product to tissue surface	35 (59%)	23 (38.9%)	1 (1.6%)
Ease of delivery to reach the wound site	37 (62.7%)	22 (37.3%)	-
Ease of preparation for use	36 (61%)	23 (39%)	-

## Discussion

This study evaluates the efficacy, performance, and safety of Surgi-ORC® as a hemostatic agent in different types of orthopedic surgeries. The findings from this study contribute valuable insights into the Surgi-ORC® performance, safety, and practical utility in real-world clinical settings. The data confirm that Surgi-ORC® is effective in achieving rapid hemostasis, with most patients achieving TTH within three minutes (average TTH: 1.95 minutes (Surgi-ORC® Original/Standard), range = 0.71 minutes (Surgi-ORC® Powder) to 2.50 minutes (Surgi-ORC® Knit)). This range of TTH is consistent with what is typically observed for advanced hemostatic agents, suggesting that Surgi-ORC® performs well in managing intraoperative bleeding across different clinical scenarios. The chi-square test found no statistically significant association between surgery and the degree of bleeding, suggesting the type of surgery performed did not significantly influence the degree of bleeding. Box plot analysis revealed the lowest median TTH and narrow interquartile range for axial skeleton surgery, indicating low variability. In contrast, lower limb surgery had the highest median TTH and wider interquartile range, suggesting higher variability and more spread in data. These results highlight the differences in TTH distribution across the three types of surgeries, with axial skeleton surgeries being the most consistent and lower limb surgeries exhibiting the greatest variability. Statistical analysis revealed that powder is the best option for achieving rapid hemostasis (less than two minutes). In the axial skeleton group, the median TTH is very low, showing that most surgeries in this category achieve hemostasis quickly. In lower limbs and upper limbs, the medians are higher, suggesting that these surgeries take longer on average to achieve hemostasis. The agent's biodegradability and bactericidal properties (pH 1-3) contribute to a reduced risk of postoperative infections and post-surgical complications, a significant advantage in orthopedic surgery. The design features of Surgi-ORC®, especially Fibril and non-woven/Snow, including its lightweight and pliable structure, enhance its adaptability to various anatomical sites and wound configurations, further supporting its efficacy and safety in clinical use. The complete absorption/resorption/degradation of Surgi-ORC® in all patients was observed on day two of postoperative follow-up, as confirmed by radiological imaging. However, the asserted timeframe for complete absorption/resorption/degradation of the Surgi-ORC® is within 28 days [16]. Upon application of the Surgi-ORC® and subsequent saturation with blood, the device initiates degradation or liquefaction once hemostasis is attained. As a result, visualizing or locating Surgi-ORC® on an X-ray may prove difficult. The capacity to identify it is contingent upon the quantity utilized and the application spot. In osseous regions with a dense structural composition, Surgi-ORC® may be discernible and identifiable by a radiologist.

Moreover, accurate identification relies on the radiologist's proficiency and acquaintance with such substances. Nonetheless, absorbable hemostatic substances may remain detectable upon placement on advanced imaging modalities such as CT scans and MRIs until complete absorption occurs [17]. Surgi-ORC® received high ratings across all safety, efficacy, and performance categories. Most surgeons found the product excellent and good in ease of application. Similarly, most users rated the conformation to the tissue surface as excellent, with only a few rating it as good and very few rating it as poor. The product was also highly rated for ease of delivery to the wound site, with the vast majority of respondents rating it as excellent and the rest as good. Lastly, ease of preparation for use received predominantly excellent ratings, with the remaining responses rating it as good. These results indicate high satisfaction with the product's performance across all evaluated characteristics. In contrast to a study by Amjadi et al. [18], the intrinsic bactericidal activity of Surgi-ORC® markedly improves safety by crucially reducing post-surgical infections, hence facilitating excellent healing results for patients. Surgi ORC® proficiently resolves issues related to cysts, localized edema, and infections commonly associated with similar treatments available in the market [19]. The safety profile of Surgi-ORC®, as evidenced by the absence of reported adverse effects in this study, aligns with existing literature on ORC products. From a safety perspective, the meticulous engineering of Surgi-ORC® with uniformity of texture ensures that it does not adhere to surgical gloves and surgical instruments. This crucial feature facilitates smooth and efficient application during surgical procedures [20]. This non-adherent characteristic is essential for maintaining a high level of dexterity and control, thereby minimizing the potential for errors during surgery. Surgi-ORC® is easy to place at the surgical site and can be safely implanted at the surgical site, and then the surgical site can be sutured. Surgi-ORC® effectively controls bleeding by forming a gelatinous matrix that facilitates platelet adhesion, promoting rapid hemostasis. This mechanism of action of Surgi-ORC® remains consistent in managing oozing and spot bleeding. The variability in blood loss observed in the study can be attributed to several factors, including the type and severity of fractures, surgical complexity, and the hemostatic techniques employed. Lower limb fractures, particularly those involving large bones like the femur or tibia, tend to result in greater blood loss compared to upper limb or axial skeleton fractures. Additionally, complex fractures requiring extensive fixation may contribute to higher intraoperative bleeding. Moreover, while Surgi-ORC® was the primary hemostatic agent used, some cases required adjunct hemostatic techniques such as electrocoagulation or mechanical methods based on the surgeon’s discretion and intraoperative bleeding severity. These variations in surgical approach and bleeding characteristics likely contributed to the broad range of blood loss observed in the study.

While this study demonstrates promising outcomes with Surgi-ORC® in achieving hemostasis, certain limitations warrant consideration. Firstly, the study was conducted on a relatively small cohort, which may limit the generalizability of the findings across diverse populations and surgical scenarios. Secondly, the lack of a randomized control group restricts the ability to compare the efficacy of Surgi-ORC® with other hemostatic agents. Finally, the absence of long-term follow-up precludes evaluation of the sustained effectiveness and potential complications associated with the product over time. Future research addressing these limitations could provide more comprehensive insights into the clinical utility of Surgi-ORC®.

## Conclusions

In conclusion, this study demonstrates the efficacy, performance, and safety of Surgi-ORC® as a hemostatic agent in orthopedic surgeries. The rapid achievement of hemostasis across various orthopedic surgeries highlights the agent's utility in managing intraoperative bleeding and enhancing positive surgical outcomes. The favorable safety profile and practical advantages of Surgi-ORC® support its integration into clinical practice for effective bleeding control in orthopedic procedures. It was observed in this study that the Surgi-ORC® Fibril was primarily preferred by surgeons in orthopedic surgery due to its lightweight and tufted texture. Surgi-ORC® Powder was found to be superior in terms of achieving rapid hemostasis in comparison to other variants of the subject device. Surgi-ORC® provides high flexibility, drapeability, and adaptability to surgical sites. It can be cut to any desired size without unraveling or fraying, implanted at the surgical site, and safely sutured without affecting the integrity or performance of the material. The device is primarily designed to achieve hemostasis across various surgical procedures, including orthopedic surgeries. Different variants of Surgi-ORC® may be used for various surgeries according to their feasibility and surgeons' experience with the device variants. However, further research is warranted to confirm these findings and to explore additional applications and improvements in hemostatic technology. 

## Figures and Tables

**Figure 6:**
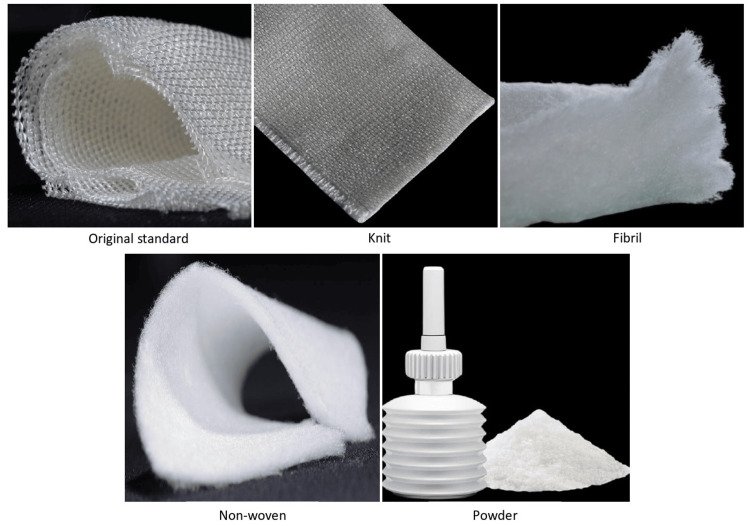
Variants of Surgi ORC®

## Appendices

Appendix A 
